# Orthologs of the small RPB8 subunit of the eukaryotic RNA polymerases are conserved in hyperthermophilic Crenarchaeota and "Korarchaeota"

**DOI:** 10.1186/1745-6150-2-38

**Published:** 2007-12-14

**Authors:** Eugene V Koonin, Kira S Makarova, James G Elkins

**Affiliations:** 1 National Center for Biotechnology Information, National Library of Medicine, National Institutes of Health, Bethesda, MD 20894, USA; 2 Microbial Ecology and Physiology Group, Biosciences Division, Oak Ridge National Laboratory, Oak Ridge, TN 37831, USA

## Abstract

Although most of the key components of the transcription apparatus, and in particular, RNA polymerase (RNAP) subunits, are conserved between archaea and eukaryotes, no archaeal homologs of the small RPB8 subunit of eukaryotic RNAP have been detected. We report that orthologs of RPB8 are encoded in all sequenced genomes of hyperthermophilic Crenarchaeota and a recently sequenced "korarchaeal" genome, but not in Euryarchaeota or the mesophilic crenarchaeon *Cenarchaeum symbiosum*. These findings suggest that all 12 core subunits of eukaryotic RNAPs were already present in the last common ancestor of the extant archaea.

This article was reviewed by Purificacion Lopez-Garcia and Chris Ponting.

## Findings

The core components of the information-processing systems, and in particular, the transcription machinery, are conserved between archaea and eukaryotes, and distinct from the bacterial versions. The heteromultimeric eukaryotic RNAPs consist of 12 subunits (Rpb1–12), of which 11 are conserved in archaea and eukaryotes whereas one, Rpb8, is thought to be unique for eukaryotes [1-6]. Rpb8 is a small protein that typically consists of ~120–150 amino acids and shows relatively poor sequence conservation in eukaryotes. The structure of Rpb8 has been solved, originally, by solution NMR [7] and, subsequently, as part of the RNAP II core, by X ray crystallography [8]. The two structures are in good agreement and indicate that Rpb8 forms a distinct version of the OB(oligonucleotide-oligosaccharide-binding) fold [9] that is characterized by a distinct pattern of 9 β-strands and a pair of invariant glycines in the turn between strands 7 and 8. In the RNAP II structure, Rpb8 interacts with the so-called pore module of Rpb1 at a defined motif that is conserved in both eukaryotic and archaeal Rpb1 orthologs [8]. In addition, Rpb8 genetically interacts with another small subunit, Rpb6, that is also adjacent to the pore module in the core RNAP structure [6]. It has been suggested that Rpb8 and Rpb6, together with the pore module, form a distinct functional unit [6]. The exact role of the small subunits remains unknown although both Rpb6 and Rpb8 are conserved in all eukaryotes, are shared between RNAP I, II and III, and are essential for yeast growth. Regardless of the precise function of Rpb8, the purported absence of this RNAP subunit ortholog in archaea is the major gap in the picture of the otherwise exact correspondence between the core transcriptional machineries of eukaryotes and archaea, and is highly unexpected considering the conservation of all other subunits including the functional partner of Rpb8, Rpb6.

In the course of the genome annotation for the first sequenced member of the "Korarchaeota", a putative deep branch of archaea ([10] and manuscript in preparation), one of us (JGE) identified a short (110 amino acids) predicted protein for which some of the best hits in a BLAST search [11] were the eukaryotic Rpb8 subunits. Although the sequence similarity was not statistically significant, this observation prompted a systematic search for possible archaeal homologs of Rpb8. BLAST searches started with the sequences of Rpb8 subunits of various eukaryotic species, again, showed statistically not significant similarity to a distinct set of crenarchaeal proteins that were similar in size to (or slightly shorter than) Rpb8. However, reciprocal iterative PSI-BLAST searches (inclusion E-value cut-off 0.01) started with some of these crenarcheal sequences showed significant similarity to Rpb8. For example, a search with the sequence from *Hyperthermus butylicus *(Hbut_0467) used as the query retrieved the Rpb8 sequence from fission yeast Schizosaccharomyces pombe in the 2^nd ^iteration, with a E-value of 0.004, and numerous eukaryotic Rpb8 sequences in the 3^rd ^iteration, with highly significant E-values. Similarly, a search with the sequence from Igniococcus hospitalis (Igni_1165) retrieved Rpb8 from Entamoeba histolytica in the 5^th ^iteration, with an E-value of 4 × 10^-5^).

**Figure 1:**
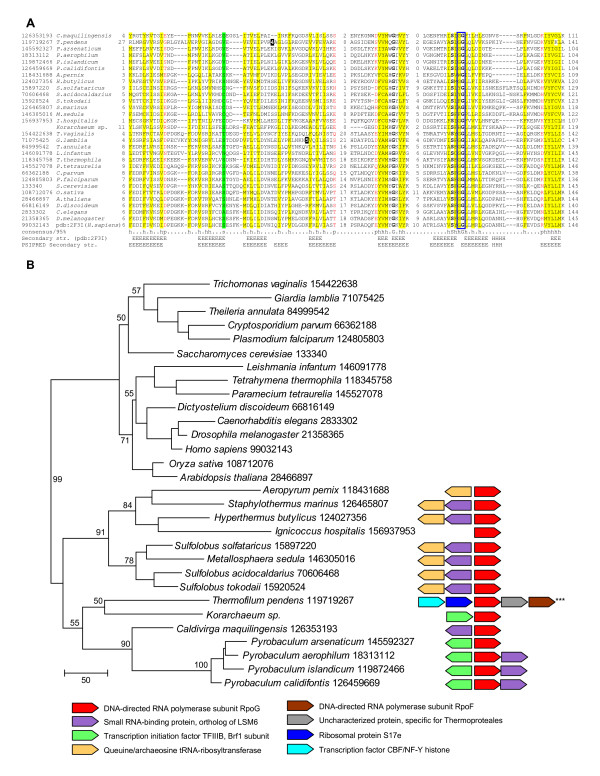
**Orthologs of Rpb8 in archaea**. (a) **Multiple alignment of eukaryotic Rpb8 subunits and their archaeal orthologs (RpoG)**. The alignment was constructed using the combination of the results obtained with PROMALS [15] and MUSCLE [16], followed by manual correction on the basis of secondary structure prediction that was obtained using PSIPRED [17] and local alignments generated by PSI-BLAST. Sequences are denoted by their numeric Genbank Identifiers (GI numbers) and species names. The full species names are given in Figure 2. The positions of the first and the last residues of the aligned region in the corresponding protein are indicated for each sequence. The numbers within the alignment represent poorly conserved inserts that are not shown. The numbers of omitted amino acids for *T. pendens *and *G. lamblia *are indicated by reverse shading. Positions with identical amino acids in all aligned sequences are in bold face. The coloring is based on the consensus shown underneath the alignment; 'h' indicates hydrophobic residues (ACFILMVWYH), 'p' indicates polar residues (STEDKRNQH), 's' indicates small residues (AGCVDS). Secondary structure is shown for the crystal structure of human Rpb8 (pdb 2F3I); 'H' indicates α-helix and 'E' indicates extended conformation (β-strand). The PSIPRED secondary structure prediction is shown underneath the experimental secondary structure. The glycine doublet that is invariant in eukaryotic Rpb8 sequences is boxed. (b) **Phylogenetic and genomic contexts of Rpb8/RpoG**. The maximum likelihood phylogenetic tree of Rpb8/RpoG was constructed by local rearrangement of an original minimum evolution (Fitch) tree [18] using the MOLPHY program [19]. MOLPHY was also used to compute RELL bootstrap probabilities, which are indicated (as percentages) for selected major branches. Each terminal node of the tree is labeled by the full species name and the GI number. The genomic neighborhoods of the *rpoG *gene in Crenarchaeota and the "korarchaeal" genome are shown to the right of the respective branches of the tree. Orthologous genes are shown by arrows of the same color.

Examination of a multiple alignment of the Rpb8 sequences from diverse eukaryotes and the putative archaeal counterparts showed remarkable conservation of all elements of the OB-fold, in particular, the diagnostic motif between strands 7 and 8 (although one of the two glycines that are invariant in Rpb8 is replaced in a subset of the putative archaeal homologs (Fig. 1a). Furthermore, secondary structure prediction for the archaeal proteins showed a near-perfect superposition with the secondary structure elements extracted from the crystal structure of Rpb8 [8] (Fig. 1a). Taken together, these findings indicate that the detected small archaeal proteins are *bona fide *homologs of Rpb8.

Small proteins homologous to Rpb8 were identified in all 10 sequenced genomes of hyperthermophilic Crenarchaeota and the only available korarchaeal genome. Each of these genomes encodes a single Rpb8 homolog which mimics the situation in eukaryotes where no paralogs of Rpb8 are detectable. By contrast, n homologs of these proteins were identified in Euryarchaeota and the mesophilic crenarchaeon *Cenarchaeum symbiosum*, despite extensive search including running a position-specific scoring matrix for Rpb8 and their crenarchaeal-korachaeal homologs against a dedicated database of euryarchaeal and *C. symbiosum *protein sequences. Thus, we conclude that all thermophilic Crenarchaeota and at least one korarchaeote encode a single ortholog of eukaryotic Rpb8 whereas Euryarchaeota and *C. symbiosum *(the only mesophilic crenarchaeon for which the genome sequence is currently available) do not.

In retrospect, we became aware that the protein we identified as the crenarchaeal ortholog of Rpb8 has already been described as one of the 13 experimentally defined subunits of the RNAP of the crenarchaeon *Sulfolobus acidocaldarius *and designated RpoG [12], and the ortholog encoded in the genome of *S. solfataricus *has been accordingly annotated [13]. Given these data, it appears sensible to adopt the designation RpoG for the archaeal orthologs of Rpb8. In a subsequent global analysis of mRNA stability in the two *Sulfolobus *species, it has been shown that the RpoG mRNA is markedly more stable than mRNAs of other RNAP subunits, and the apparent uniqueness of this subunit in *Sulfolobus *has been emphasized [14]. The present analysis clarifies the situation by showing that RpoG is conserved throughout the hyperthermophilic Crenarchaeota (and at least one korarchaeote).

Although the small size of Rpb8 and its archaeal orthologs (RpoG) hampers reliable phylogenetic analysis, the maximum likelihood tree we constructed shows a clear separation of the eukaryotic and crenarchaeal branches, and within the latter, the split between Thermoproteales and Sulfolobales (Fig. 1b). Interestingly, the korarchaeal RpoG clustered with Thermoproteales in a strongly supported branch (Fig. 1b). Broader implications of this observation for the evolution of the "Korarchaeota" remain to be investigated.

In the archaeal genomes, the *rpoG *gene is embedded in a notable, partially conserved genomic context (Fig. 1b). With few exceptions, *rpoG *forms either a codirectional or a divergent but potentially coregulated gene pair with a gene encoding a small RNA-binding protein (COG1958) that is orthologous to eukaryotic Lsm6 and is implicated in RNA-processing. In most of the Sulfolobales, the latter gene is adjacent to a gene for a tRNA modification enzyme, queuine/archaeosine tRNA-ribosyltransferase. Another common neighbor of *rpoG *is the gene for transcription elongation factor TFIIIB, with a divergent orientation in the majority of Thermoproteales and a codirectional orientation in the korarchaeote. Finally, *Thermofilum pendens *appears to have a more complex operon organization, with the genes for another RNAP subunit, a transcription factor and a ribosomal protein in the same predicted operon with *rpoG *(Fig. 1b). This genomic context suggests that, in Crenarchaeota, RpoG is likely to be involved in a tight functional cooperation with TFIIIB, and could also contribute to coupling transcription with RNA processing and modification.

The finding described here fills the last gap in the one-to-one correspondence between the RNAP subunits of archaea and eukaryotes, with the implication that the archaeal "parent" of eukaryotes already possessed the intricate 12-subunit organization of RNAP. Surprisingly, however, Euryarchaeota and the only available genome of a mesophilic crenarchaeon appear to lack an ortholog of Rpb8, a conclusion that is compatible with the report on the reconstruction of a fully active RNAP of the euryarchaeon *Methanocaldococcus jannaschii *from 12 recombinant proteins which, obviously, did not include Rpb8 [1]. Depending on the adopted evolutionary scenario, it is conceivable that Rpb8 emerged in the crenarchaeal lineage or, perhaps, more plausibly, that it was already present in the common ancestor of all extant archaea but lost at the base of the euryarchaeal branch. Regardless of the solution to this conundrum, experimental study of functional differences between RNAPs of Euryarchaeota and Crenarchaeota should be illuminating, given the unusual difference in their predicted subunit composition.

## Abbreviations

RNAP: RNA polymerase.

## Competing interests

The author(s) declare that they have no competing interests.

## Reviewers' reports

Reviewer 1: Purificacion Lopez-Garcia, Universite Paris-Sud

This manuscript reports the identification of genes orthologous to RPB8 in archaea. This observation is very interesting and worth publishing, as RPB8 was the only protein conserved in the eukaryotic core of RNA polymerases I, II and III for which orthologues in archaea had not been found. The fact that this protein is apparently missing from Euryarchaeota is intriguing, suggesting that either its role can be fulfilled by other elements or that its primary sequence has evolved beyond recognition. At any rate, the finding of archaeal RPB8 homologues indicates that the complete RNA polymerase core in both archaea and eukaryotes share a common ancestry.

Reviewer 2: Chris Ponting, Oxford University

This manuscript demonstrates convincingly the presence of an orthologue of eukaryotic RPB8 in hyperthermophilic Crenarchaeota. As such, it provides the last missing piece in the RNAP "puzzle" and should thus be of interest to many working in this field.

## Authors' contributions

EVK contributed to sequence analysis and wrote the manuscript; KSm contributed to sequence analysis; JGE made the original observation on the presence of an Rpb8 homolog among the "korarchaeal" proteins; all authors read, edited and approved the final version of the manuscript.
